# Supplementary figures and images for: Enriched atlas of lncRNA and protein-coding genes for the GRCg7b chicken assembly and its functional annotation across 47 tissues
Source: Sci Rep. 2024 Mar 19;14:6588. doi: 10.1038/s41598-024-56705-y (PMC10951430; doi:10.1038/s41598-024-56705-y)

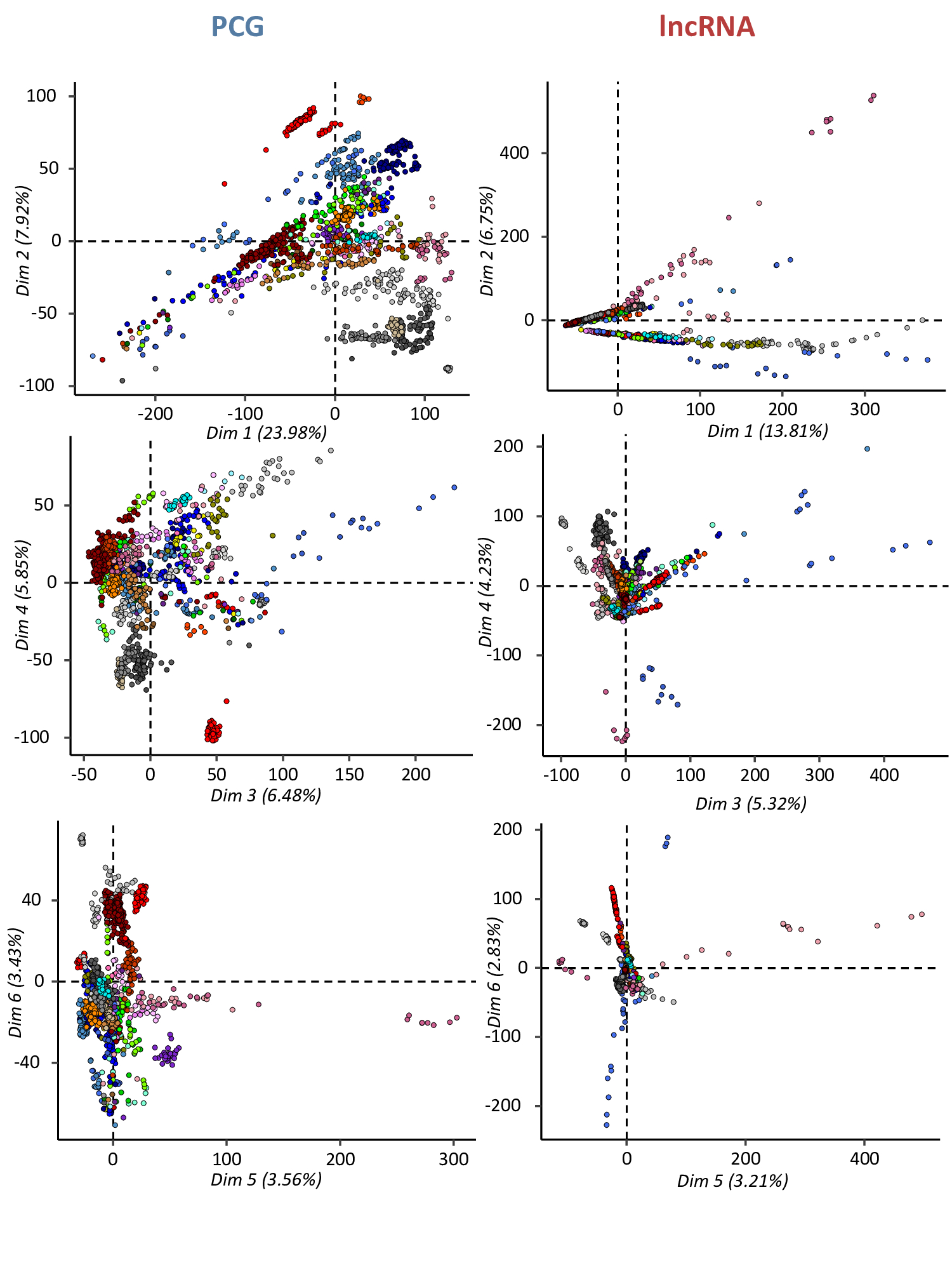

Supplement: Supplementary file 1 — Supplementary Figure 1. [file 41598_2024_56705_MOESM1_ESM.jpg]

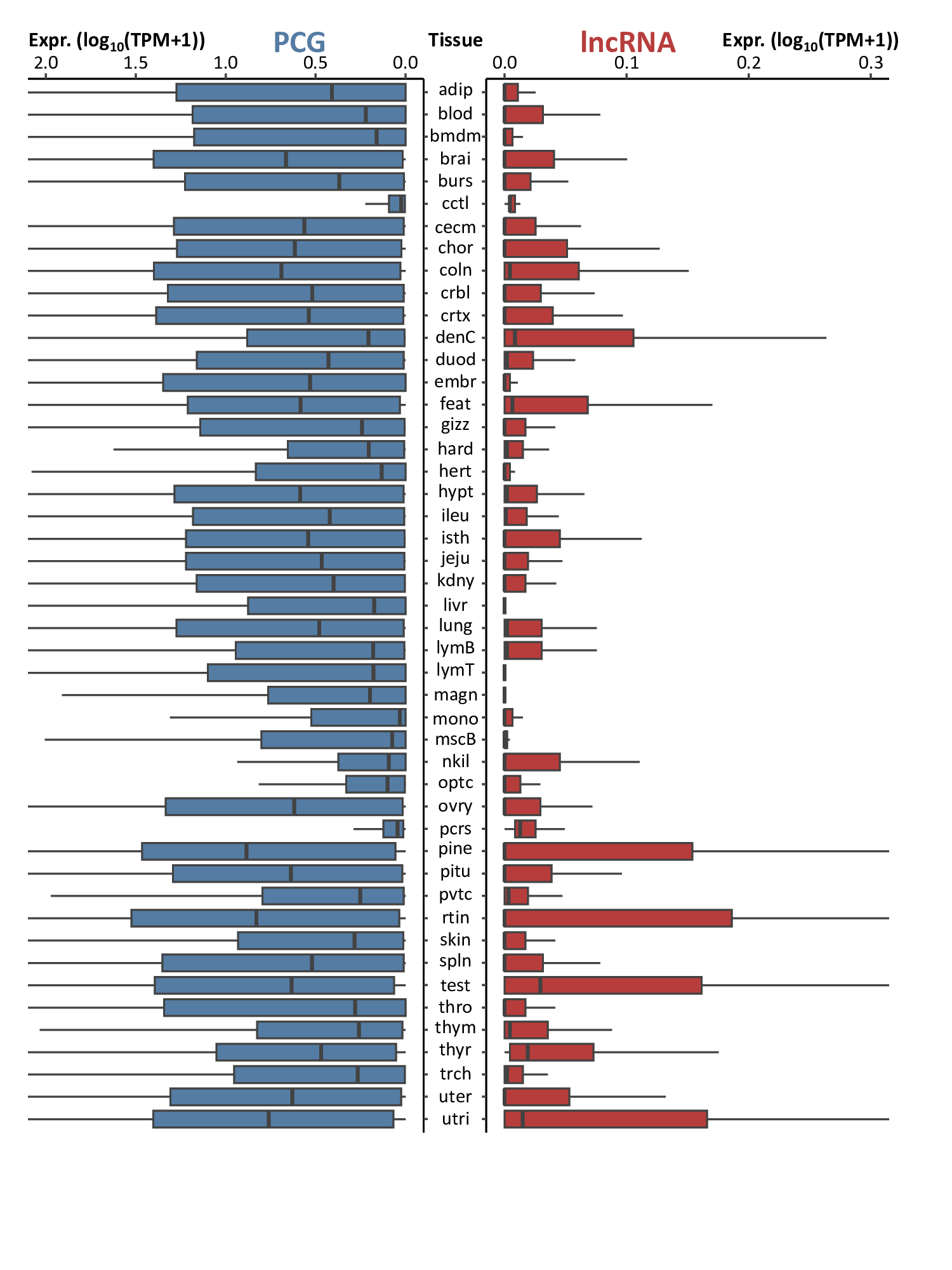

Supplement: Supplementary file 2 — Supplementary Figure 2. [file 41598_2024_56705_MOESM2_ESM.jpg]

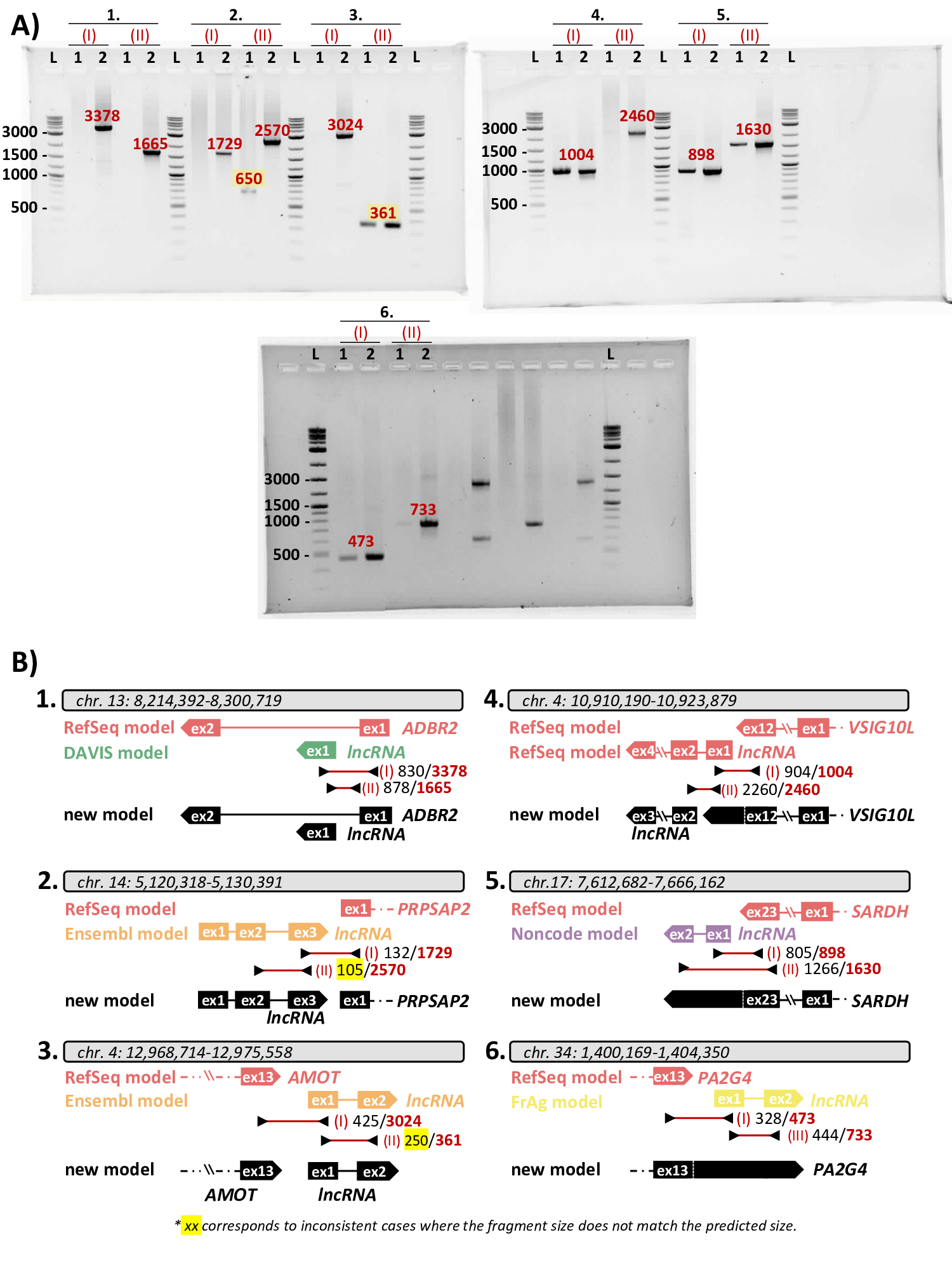

Supplement: Supplementary file 3 — Supplementary Figure 3. [file 41598_2024_56705_MOESM3_ESM.jpg]
