# Supplementary material for: Enriched atlas of lncRNA and protein-coding genes for the GRCg7b chicken assembly and its functional annotation across 47 tissues
Source: Sci Rep. 2024 Mar 19;14:6588. doi: 10.1038/s41598-024-56705-y (PMC10951430; doi:10.1038/s41598-024-56705-y)
Supplement: Supplementary file 21 — Supplementary Legends. [file 41598_2024_56705_MOESM21_ESM.docx]

SUPPLEMENTARY FIGURES

**Sup. Figure 1.** Principal component analysis based on gene expression of expressed PCGs and lncRNAs.

The factorial plans for axes 1:2, 3:4 and 5:6 are provided. Colours and associated tissues are available in Sup. Table 15.

**Sup. Figure 2.** Distribution of PCG (blue) and lncRNA gene expression in log_10_(TPM+1) in chicken for the 47 tissues.

Full tissue names for chicken are available in Sup. Table 15.

**Sup. Figure 3.** Reliability of six lncRNAs in same-strand configuration of a PCG tested by PCR. a) Resulting gels for each pair tested. The first line of indication above each gel corresponds to: L: ladder; 1: PCR using cDNA; 2: PCR using genomic DNA (gDNA). The second line with roman numerals refers to the PCR primer pairs used, which are indicated in the part b) with the predicted size for cDNA and gDNA. The third line indicate the lncRNA:PCG pair tested. Red values correspond to the observed size of the amplified fragment compared with the predicted size. b) Models of lncRNA:PCG pair tested. The upper part of each panel (in color) represents the relative position of the constituent genes of the lncRNA:PCG pair as identified on the enriched atlas. The lower panel (black) shows the constituent genes of the lncRNA:PCG pair based on the PCR results. Left: lncRNAs considered as independent loci from the (1) DAVISGALG000044072/ADBR2, (2) ENSGALG00010022678/PRPSAP2, and (3) ENSGALG00010016012/AMOT lncRNA:PCG pairs. Right: lncRNAs considered as extension of the PCG from the (4) LOC121113202/VSIG10L, (5) NONGGAG001811/SARDH, and (6) FRAGALG000000006896/PA2G4 lncRNA:PCG pairs.

SUPPLEMENTARY TABLES

**Sup. Table 1.** Gene annotation with genomic and functional information/features for gene models of the enriched‑atlas including the orthology, the expression, the tissue‑specificity, the classification of gene models with the closest PCG or lncRNA, GO terms but also identifiers equivalence between the two reference genome annotations “RefSeq” and “Ensembl”. Also available at [www.fragencode.org/lnchickenatlas.html](http://www.fragencode.org/lnchickenatlas.html) with the corresponding genome annotation (.*gtf*).

**Sup. Table 2.** Genome annotation in BigBed format for use with browsers.

**Sup. Table 3.** Gene model sequence in FASTA format with header indicating ">(Strand)Gene_id::::Chromosome:Start-End". All sequences are provided according to the sense DNA strand.

**Sup. Table 4.** Transcript model sequence in FASTA format with header indicating ">(Strand)Gene_id: Transcript_id:::Chromosome:Start-End". All sequences are provided according to the sense DNA strand.

**Sup. Table 5.** Exon model sequence in FASTA format with header indicating ">(Strand)Gene_id: Transcript_id: Exon_id::Chromosome:Start-End". All sequences are provided according to the sense DNA strand.

**Sup. Table 6.** Characteristics of the gene models included in each genome annotation used to build the enriched‑annotation. (a) Size and number of genes, transcripts, exons and their associated proportions for lncRNAs, PCGs and all gene models. (b) Number of lncRNAs and PCGs supported by one (“1tr”) or more (“Xtr”) transcripts and with one (“1ex”) or more (“Xex”) exons. Transcripts classified as multi‑exonic but with only one exon longer than 50bp are considered as “False Multi­‑ exonic” ("FM"). (c) Number and types of biotypes indicated in each database.

**Sup. Table 7.** Number of genes and their associated biotypes successively added per database used to build the enriched‑annotation.

**Sup. Table 8.** Project accession numbers and number of samples used to quantify the gene expression across the 47 tissues composing the atlas.

**Sup. Table 9.** Number of expressed and tissue­­-specific PCGs and lncRNAs across the 47 tissues for an expression threshold of 0.1 and 1 TPM. mono_TS: genes specific to a single tissue, poly2to7_TS and poly8to47_TS: genes specific to a group of n tissues with n ≤ 7 and n > 7 respectively. Full tissue names for chicken are available in Sup. Table 11.

**Sup. Table 10.** Genes related to a known Mendelian trait or disorder (“Phene”) obtained from the OMIA resource. The hypothetical tissue in which the causative gene/variant is likely to have an effect is indicated in the "ExpectedTissue" column. For each gene, its name (“GeneName”), its genes identifier in “RefSeq” (“GeneId”) and in “Ensembl” both by BioMart (“GeneId_BiomartEnsEq”) and by overlap (“GeneId_OvlpEnsEq”) are provided according to the GRCg7b assembly.

**Sup. Table 11.** List of differentially expressed genes (DEGs) between sexes (male/female) detected in the liver (livr), adipose tissue (adip), bone marrow‑derived macrophages (bmdm), bursa of Fabricius (burs), feather (feat), and the Harderian gland (hard). For “bmdm”, the analysis was conducted on two independent projects and the union of DEGs was used. For each gene, its name (“GeneName”), its genes identifier in “RefSeq” (“GeneId”) and in “Ensembl” both by BioMart (“GeneId_BiomartEnsEq”) and by overlap (“GeneId_OvlpEnsEq”) are provided according to the GRCg7b assembly.

**Sup. Table 12.** Equivalence table of the gene identifiers from our previous annotation in galgal5 and GRCg6a to the one in GRCg7b. Two types of list are provided: *i)* an equivalence gene by gene with the coordinates in both assembly; *ii)* an equivalence only with gene identifiers collapsed considering GRCg7b as the reference.

**Sup. Table 13.** Numbers of lncRNAs and PCGs according to their configuration with their closest PCG and their genome annotation origin.

**Sup. Table 14.** Priorization of the gene biotypes applied when gathering the different genome annotations.

**Sup. Table 15.** Names, abbreviations and colours of the 47 chicken tissues.

**Sup. Table 16.** Names, abbreviations and colours of the 53 human GTEx tissues.

**Sup. Table 17.** Primers sequences and corresponding annealing temperature used for PCR analysis of lncRNA:PCG pairs in same strand configuration.
